# Development of replication-competent adenovirus for bladder cancer by controlling adenovirus E1a and E4 gene expression with the survivin promoter

**DOI:** 10.18632/oncotarget.2151

**Published:** 2014-06-30

**Authors:** Ho Kyung Seo, Jeong Bin Seo, Jae-Kook Nam, Kyung-Chae Jeong, Seung-Pil Shin, In-Hoo Kim, Sang Don Lee, Sang-Jin Lee

**Affiliations:** ^1^ Center for Prostate Cancer, Gyeonggi-do, Biomedical Research Institute, Pusan National University and Research Institute for Convergence of Biomedical Science and Technology, Pusan; ^2^ Pusan National University and Biomedical Research Institute, Pusan National University and Research Institute for Convergence of Biomedical Science and Technology, Pusan; ^3^ Genitourinary Cancer Branch, Research Institute National Cancer Center, Gyeonggi-do, Korea; ^4^ Biomolecular Function Research Branch, Research Institute National Cancer Center, Gyeonggi-do, Korea; ^5^ Molecular Imaging and Therapy Branch, Research Institute National Cancer Center, Gyeonggi-do, Korea

**Keywords:** bladder cancer, gene therapy, adenovirus, survivin

## Abstract

Survivin is a member of the inhibitors of apoptosis protein family. Here, we examined survivin expression and confirmed abundant survivin expression in bladder cancer cells. This expression pattern indicated that the transcriptional regulatory elements that control survivin expression could be utilized to discriminate cancer from normal cells. We therefore generated a novel adenovirus termed Ad5/35E1apsurvivinE4 with the following characteristics: 1) E1A and E4 protein expression was dependent on survivin promoter activity; 2) the green fluorescence protein gene was inserted into the genome under the control of the CMV promoter; 3) most of the E3 sequences were deleted, but the construct was still capable of expressing the adenovirus death protein with potent cytotoxic effects; and 4) the fiber knob was from serotype 35 adenovirus. As expected from the abundant survivin expression observed in bladder cancer cells, Ad5/35E1apsurvivinE4 replicated better in cancer cells than in normal cells by a factor of 10^6^ to 10^2^. Likewise, Ad5/35E1apsurvivinE4 exerted greater cytotoxic effects on all bladder cancer cell lines tested. Importantly, Ad5/35E1apsurvivinE4 inhibited the growth of Ku7-Luc orthotopic xenografts in nude mice. Taken together, Ad5/35E1apsurvivinE4 indicates that the survivin promoter may be utilized for the development of a replication-competent adenovirus to target bladder cancers.

## INTRODUCTION

Urothelial carcinoma of the bladder is the second most common genitourinary malignancy and the second most common cause of genitourinary cancer-related death [1]. In particular, 70 to 80% of patients with urothelial carcinoma of the bladder present with non-muscle invasive bladder cancer (NMIBC). The standard treatment for NMIBC is transurethral resection (TUR) with or without intravesical therapy; however, approximately 70% of patients develop recurrence after initial treatment, with up to 30% of patients progressing to muscle-invasive disease.

Radical cystectomy with urinary tract reconstruction provides for the optimal control of high-risk NMIBC and muscle-invasive bladder cancer. Although advances in reconstruction techniques of the lower urinary tract have decreased the lifestyle changes associated with radical cystectomy, significant quality of life alterations occur after radical surgery [2-7].

Gene therapy has garnered significant attention as a therapeutic approach for bladder cancer. Specifically, the intravesical route has demonstrated significant advantages because the virus comes in direct contact with the bladder cancer and eliminates the need for systemic administration. Avoidance of the systemic circulation minimizes the immune system's clearance of the virus and avoids viral elimination via the liver. However, efficient delivery and selectivity remain major hurdles to this type of treatment.

Survivin, which spans 14.7 kb on chromosome 17q25 and consists of three introns and four exons, is a member of the inhibitors of apoptosis protein (IAP) family [8]. This 16.6 kDa protein is characterized by a single Baculovirus IAP repeat (BIR) domain and an extended carboxy-terminal α-helical domain [9]. However, unlike other IAP family members, survivin does not contain a RING finger domain. Moreover, its expression, which is regulated by a TATA-less promoter and a GC-rich region upstream of exon 1 [10], is markedly increased in the G_2_M phase of the cell cycle [11]. During this phase, survivin protein in the nucleus is bound to and phosphorylated by p34^cdc2^/cyclin B1 kinase [12], and the phosphorylated form of survivin plays a critical role in maintaining the mitotic apparatus that enables normal mitotic progression. Survivin expression is very abundant in the vast majority of cancers, including esophageal, lung, ovarian, central nervous system, breast, colorectal, bladder, gastric, prostate, pancreatic, laryngeal, uterine, hepatocellular, and renal cancers [13].

In bladder cancer, the urinary levels of the survivin gene at both the protein and mRNA levels have been shown to be associated with cancer presence, higher tumor grade, advanced pathologic stage, and resistance to radiation and chemotherapy [14-17]. As a result, tremendous efforts have been made to counteract survivin activities in cancer cells through the utilization of antisense oligonucleotides [18-23], ribozymes [24-26], small interfering RNA [27, 28], and dominant negative survivin [29-31]. In addition, survivin may provide a new target for cancer therapies that could discriminate between transformed and normal cells. The promoter that controls survivin expression has been utilized to construct replication-competent adenovirus for targeting high survivin-expressing tumor cells [32, 33]. In this study, the survivin promoter was utilized to develop a replication-competent adenovirus.

Commonly used adenovirus vectors are composed of adenovirus serotype 5 (Ad5), and the Coxsackievirus adenovirus receptor (CAR) plays a crucial role in Ad5. Cancer cells with decreased expression of CAR are resistant to viral infection and, therefore, to adenovirus-mediated gene therapy [34]. However, bladder cancer cell lines and tumors, especially those at an advanced state and high grade, frequently lose CAR expression, which limits the use of oncolytic adenoviruses to treat bladder cancer. To overcome the limitations of Ad5 vectors, we used a chimeric recombinant adenovirus that was composed of the serotype 5 backbone and included the fiber knob from adenovirus serotype 35 (Ad35). Ad35 vectors recognize human CD46, not CAR, as the cellular receptor for infection; because human CD46 is expressed in almost all human cells, Ad35 vectors demonstrate broad tropism to human cells. The recombinant adenovirus that we generated contained the serotype 35 fiber knob, and its E1A and E4 protein expression was controlled by the survivin promoter.

## RESULTS

### Survivin expression in bladder cancer

**Figure 1:**
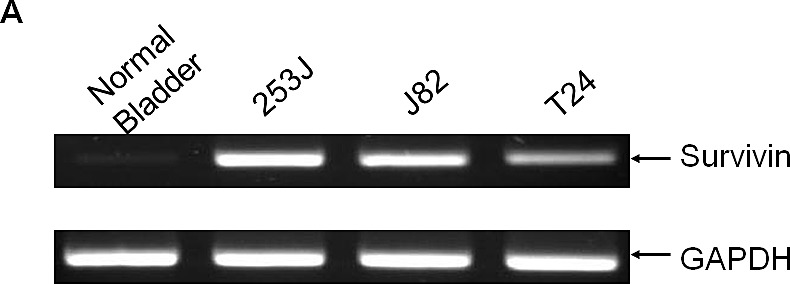

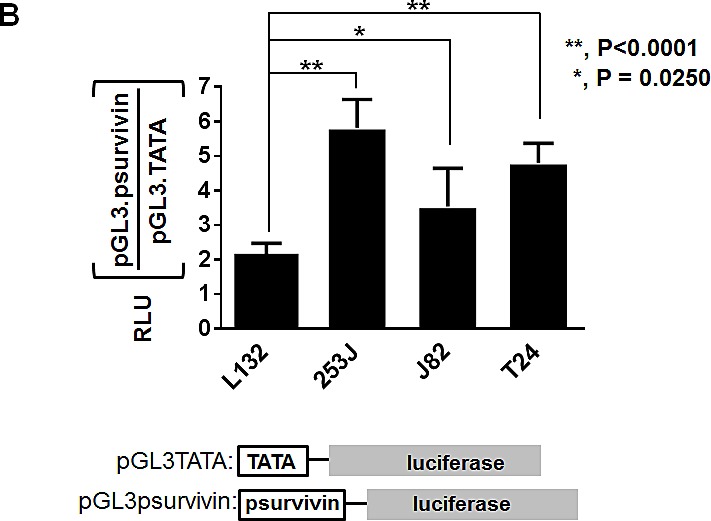
Survivin is highly expressed in all bladder cancer cells A. Total RNA was prepared from each cell line and from a normal bladder. Total RNA (1 μg) was reverse-transcribed, and PCR was performed to detect survivin expression. GAPDH was included as an internal control. B. Each bladder cell line was transiently co-transfected with 750 ng of the survivin promoter reporter construct pGL3survivin.Luc and 75 ng of pSV40.renillaLuc for 24 h. Dual luciferase activity was measured in the cell lysates and normalized to that of pGL3TATA. Data represent the mean ± SD.

All of the bladder cell lines tested showed high levels of survivin mRNA transcripts by PCR (shown in Figure 1). In contrast, total RNA prepared from normal bladder tissue did not show survivin expression. The promoter driving survivin expression in bladder cancer cells was then evaluated using reporter constructs in which the promoter was placed upstream of the luciferase gene. Following transfection into bladder cells, the survivin promoter (psurvivin) exhibited a 3.5- to 5.8-fold higher transcriptional activity compared to basic transcription driven by a TATA box. These results led us to explore whether the regulatory elements mediating survivin expression may be utilized to construct a replication-competent adenovirus under the control of the survivin promoter.

### Ad5/35E1apsurvivinE4 adenovirus construction

**Figure 2:**
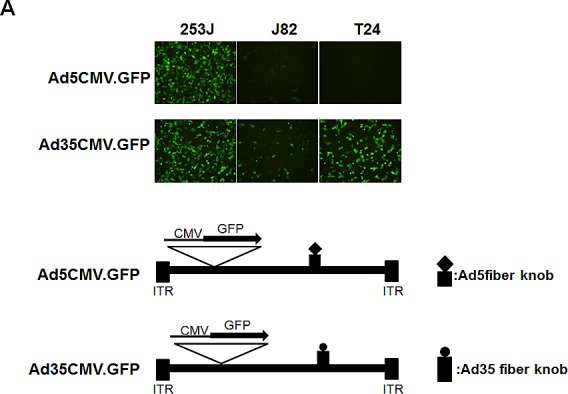

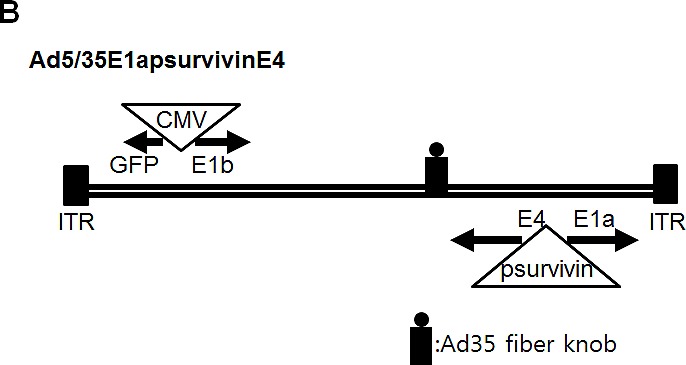
Construction of a replication-competent adenovirus with controlled survivin promoter expression A. Enhanced transduction efficiency of Ad5/35 compared to Ad5. Each bladder cell line was infected with Ad5GFP or Ad35GFP at 5 MOI. Forty-eight hours later, the GFP-positive cells were analyzed by fluorescence microscopy. B. The replication of adenovirus Ad5E1apsurvivinE4 was controlled by the survivin promoter. The organization of the adenoviral early transcripts (E1A, E1B, and E4) is illustrated schematically. The enhanced GFP gene was inserted to monitor adenovirus activity.

Most adenoviral backbones used for gene therapy are based on adenovirus serotype 5. Infection with this serotype requires high-affinity interactions between its viral fiber and a cellular CAR. Although adenovirus efficiently infects a large variety of human tumor cell lines, the transduction of solid tumors is often limited by the low level of CAR expression, which results in low infectivity in highly aggressive tumors. For this study, we determined whether the fiber knob of serotype 35 could enhance the adenoviral infectivity to bladder cancer cells. Bladder cancer cells were therefore infected with adenovirus serotypes 35 and 5, which enabled delivery of the gene encoding green fluorescence protein (GFP) under control of the CMV promoter. GFP fluorescence was monitored 2 days post-infection using fluorescence microscopy. As shown in Figure 2A, serotype 35, which utilizes CD46 for its receptor on target cell surfaces, exhibited greater efficiency in T24 and J82 cells and was therefore used to construct a chimeric recombinant adenovirus. We replaced the chimeric adenovirus containing the serotype 5 backbone with the fiber knob from serotype 35. To control adenoviral replication in high survivin-expressing cancer cells, we inserted the survivin promoter into the same backbone as was previously used [11]. In this construct, we placed the adenoviral *E1A* gene at the right end of the adenoviral genome to avoid potential interference from the adenoviral packaging signal and placed the survivin promoter between the *E1A* and *E4* genes to control their expression. A CMV promoter-controlled EGFP expression cassette was inserted at the left end of the adenoviral genome to enable us to monitor viral propagation *in vitro* and virus distribution *in vivo*.

### Ad5/35E1apsurvivinE4 activity is correlated with survivin expression

**Figure 3:**
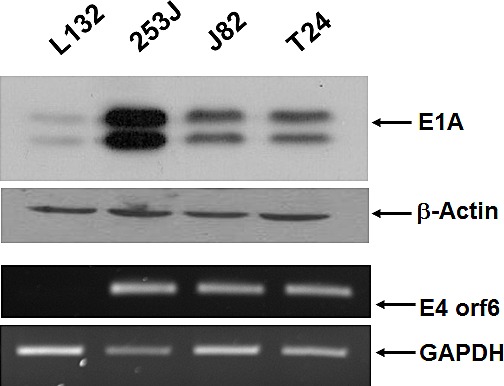
E1A and E4orf6 expression is mediated by Ad5E1psurvivinE4 Cells were seeded in 6-well plates and infected with adenovirus. Total cell lysates were prepared 24 h after viral infection, and E1A expression was then determined using anti-E1A reactive antibodies. β-actin was included as a control. Total RNA was prepared from each of the infected cell lines. Total RNA (1 μg) was reverse-transcribed, and PCR was performed to quantitate the level of E4orf6 transcript. GAPDH was included as an internal control.

To test whether the survivin promoter controlled E1A protein expression in bladder cancer cells, we infected T24, J82, and 253J cells with either Ad5/35E1apsurvivinE4 or the wild-type serotype 35 adenovirus. Human lung epithelial L132 cells were included as a survivin-negative cell line, and wild-type adenovirus was included as a positive control. One day after viral infection, E1A protein expression was examined in all of the cell lines using western blot analysis (Figure 3). Consistent with the promoter activity of psurvivin, 253J cells exhibited the largest amount of E1A protein. Expression in L132 cells was almost as low as the basal level observed in non-infected cells. Because of the lack of availability of E4-specific antibodies, reverse-transcription PCR was used to compare the expression profiles of E4 mRNA among several human bladder cancer cells and normal lung L132 cells (Figure 3). Ad5/35E1apsurvivinE4 mediated the expression of high amounts of E4 mRNA in all of the bladder cancer cells tested but not in normal lung epithelial cells. Taken together, Ad5/35E1apsurvivinE4-mediated E1A and E4 expression was well correlated with survivin expression.

**Figure 4:**
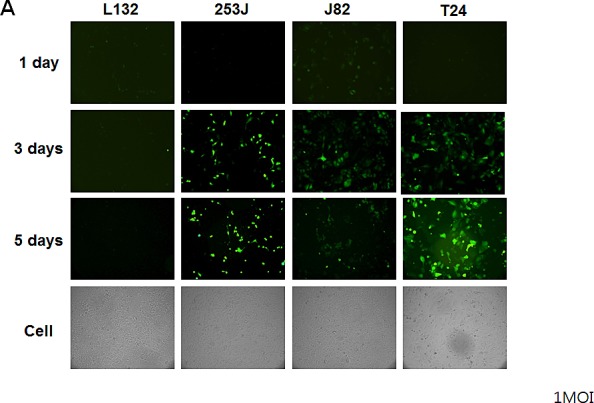

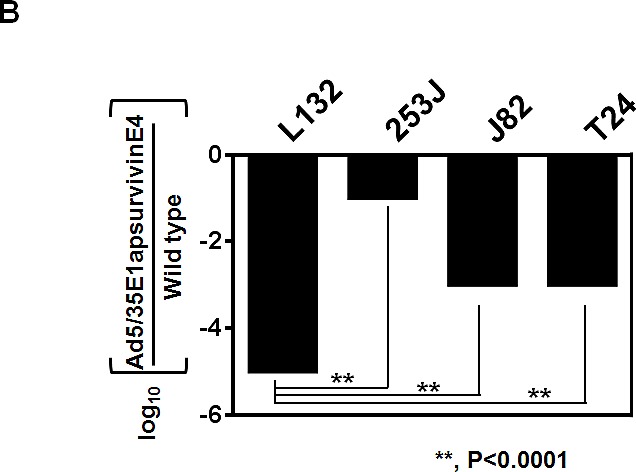
Restrictive replication of Ad5/35E1apsurvivinE4 in survivin-positive cells A. Ad5/35E1apsurvivinE4 (at 1 MOI) was added to 96-well plates containing each bladder cell line and the L132 cell line, as a negative control. Cells were monitored daily by fluorescence microscopy. B. Cells (0.5 x10^6^) were seeded in 6-well plates for 24 h and then exposed to the viruses. Twenty-four hours after infection, the cells were washed with PBS to remove the viruses. Two days later, the adenoviruses from the supernatants and cell pellets were harvested, and the numbers of adenovirus particles were quantified as described in the Materials and Methods.

To investigate the replication of Ad5/35E1apsurvivinE4, we infected bladder cancer cells with the Ad5/35E1apsurvivinE4 virus and then monitored the infected cells for up to 5 days under a fluorescent microscope (Figure 4A). Three days after infection, GFP-expressing cells became visible in all of the cancer cell types that were tested except for the L132 cells. We were also able to detect viral plaques using a light microscope. These results demonstrated that the survivin promoter was able to control the replication of Ad5/35E1apsurvivinE4 in survivin-positive cancer cells. To quantify viral replication in the bladder cancer cells, we next investigated the replication efficiency of Ad5E1apsurvivinE4 and compared it to that of the wild-type adenovirus. Five days after infection with Ad5/35E1apsurvivinE4 or wild-type adenovirus, cells and supernatants were harvested, and virus particles were released by three freeze-thaw cycles. The collected viral particles were quantified as described in the Materials and Methods section. As shown in Figure 4B, Ad5E1apsurvivinE4 replication was 10- to 1,000-fold higher in survivin-positive bladder cancer cells compared to survivin-negative L132 cells.

### *In vitro* toxicity assay

**Figure 5:**
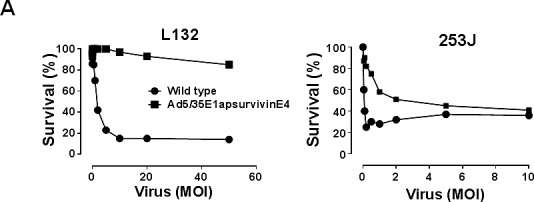

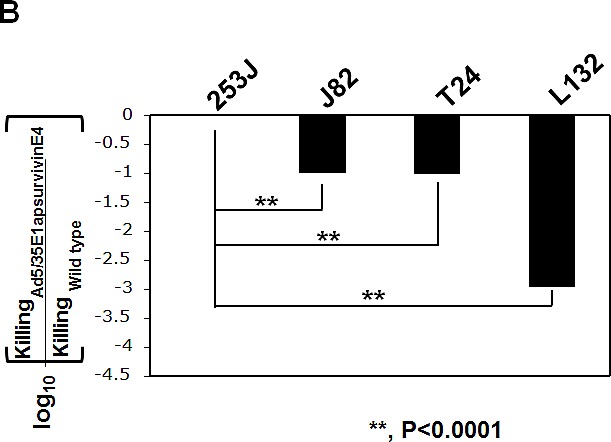
*In vivo* cytotoxic activity of Ad5/35E1apsurvivinE4 A. Each cell line (3 × 10^3^ cells/well) was plated in a 96-well plate, and cell survival was monitored in the presence of various amounts of adenovirus. MTT assays were used to count viable cells after exposure to Ad5E1apsurvivinE4 for 48 hours. B. Ad5E1apsurvivinE4 and wild-type adenoviruses were added to each of the bladder cell lines and to the L132 cell line, as a negative control. The virus doses were identical in the first row (5 pfu/cell). Cells were monitored daily using a microscope. The viral killing activity was obtained by dividing the LD50 value of the therapeutic viruses by that of the wild-type virus.

We next investigated whether the adenovirus killed the cells because of viral replication. Each of the bladder cancer cell lines was plated at a density of 1 × 10^4^ cells/well in a 96-well plate, and the cells were then exposed to various concentrations of adenovirus. Following incubation for 48 h, cell survival was determined with an MTT assay. When 253J cells were incubated for 48 h, the cytotoxic activity reached 50% at a multiplicity of infection (MOI) of 2 (Figure 5A). In contrast, the proliferation of survivin-negative human L132 cells was not significantly affected by the Ad5E1apsurvivinE4 adenovirus. Wild-type adenovirus killed both L132 and 253J cells with high efficiency. To test for the tumor-specific killing activity of Ad5/35E1apsurvivinE4, serial dilutions of Ad5/35E1apsurvivinE4 and wild-type adenovirus were applied to each cell line in 96-well plates (Figure 5B), and cell number and cell status were monitored under the microscope every day following infection. After normalization to the results obtained for wild-type adenovirus, we determined the viral titers that caused a cytopathic effect in at least four wells of each cell line in the 96-well plates. Ad5/35E1apsurvivinE4 was able to kill all of the cancer cells, although the killing potency was 10- to 100-fold lower than that of the wild-type adenovirus; these results are summarized in Figure 5B. In addition, the replication-competent adenovirus was 100-fold better at causing cell lysis in T24 and J82 cells compared to survivin-negative L132 cells. This result indicates that the killing activity of Ad5E1apsurvivinE4 was more damaging to the cancer cell lines than to L132 cells.

### Tumor suppression of survivin-positive tumors in an orthotopic murine model

**Figure 6:**
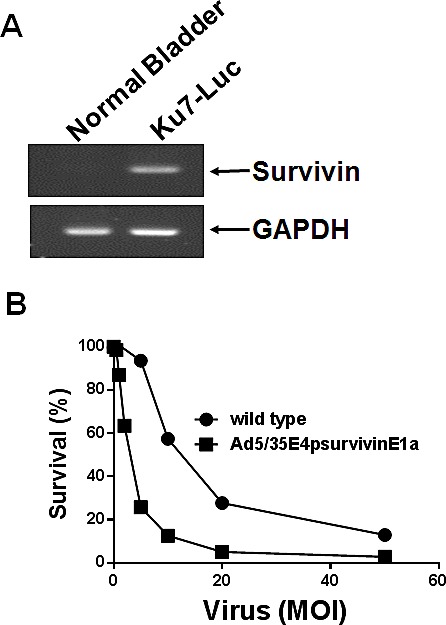

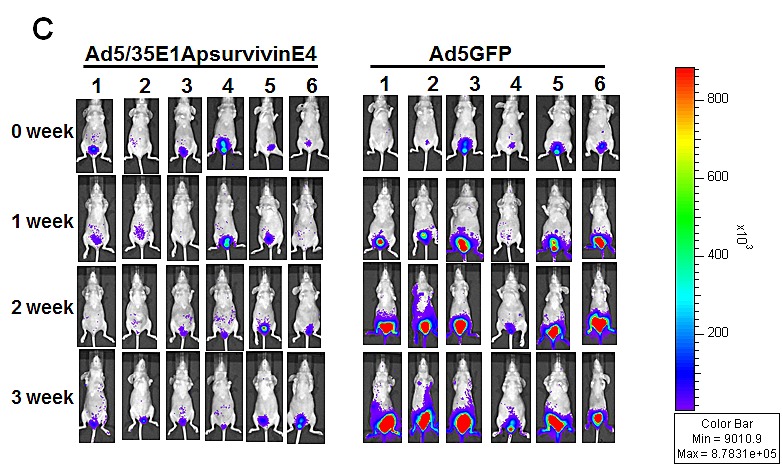
Tumor growth suppression mediated by Ad5/35E1apsurvivinE4 A. Total RNA was prepared from Ku7-Luc cells. PCR was performed to detect survivin expression, and GAPDH was included as an internal control. B. Ku7-Luc cells were plated and exposed to different concentrations of adenoviruses. Cell survival was measured using MTT assays. C. Ku7-Luc orthotopic xenografts were established in the bladders of athymic nude mice. Tumors were treated with either Ad5E1apsurvivinE4 or Ad5CMVGFP through a catheter, and tumor growth was monitored every three days using an *in vivo* imaging instrument.

Among the available orthotopic bladder murine models, we selected Ku7-Luc cells. Prior to evaluation of the tumor suppressive activity of Ad5/35E1apsurvivinE4, survivin expression in Ku7-Luc cells was determined (Figure 6A). As demonstrated by RT-PCR analysis in Figure 6, Ku7-Luc cells expressed a significant amount of survivin mRNA transcript, which indicated that the survivin promoter activity was high enough to be sensitive to lysis by Ad5E1apsurvivinE4 replication. To confirm the cytotoxic effects, Ku7-Luc cells were exposed to different doses of Ad5E1apsurvivinE4, and then viable cells were quantitated using an MTT assay (Figure 6B). As shown in Figure 6B, 2 MOI of Ad5E1apsurvivinE4 killed almost 70% of the Ku7-Luc cells, and this effect was less than that observed with wild-type adenovirus. Then, Ku7-Luc orthotopic bladder tumors were established, and 5 × 10^8^ plaque-forming units (pfu) were used to evaluate the tumor suppression efficacy. Ad5E1apsurvivinE4 was introduced into the bladder through a catheter twice per week for two weeks. Direct luminescence was then captured with an *in vivo* luminescence instrument (Figure 6C). As a result of instillation of the Ad5E1apsurvivinE4 adenovirus, Ku7-Luc tumor growth was dramatically suppressed compared to the Ad5GFP control group. These *in vivo* results demonstrate that this replication-competent adenovirus may be useful for further development.

## DISCUSSION

The clinical application of adenovirus-mediated gene therapies for cancer patients involves the following goals: 1) to establish a normal cell cycle and subsequent apoptosis by delivering tumor suppressor genes such as p53; 2) to overexpress toxic genes that target cancer cells, such as PSA-driven herpes simplex virus thymidine kinase; 3) to enhance host anti-cancer immunity by overexpressing immune modulators; and 4) to induce cell lysis by virus amplification with or without the expression of therapeutic gene(s). Recent work has focused on controlling virus replication inside of tumors. The use of replicating adenoviruses has been shown to be a promising method for treating bladder cancer patients [35-38]. In particular, the lytic process of the viral life cycle can cause cell death in a directed fashion when the *E1A* and *E1B* genes, which are key regulators of viral replication, are modified to contain a tumor-specific promoter [35, 37] or lack a functional *E1B* sequence [36].

All adenoviruses with double-stranded DNA genomes of approximately 38 Kb are encapsulated by three major proteins, including the hexon, penton, and fiber knob proteins. Following the treatment of 30 cervical cancer patients with different serotypes in the 1950s, genetic engineering technologies have improved to allow for the selective replication of viruses in tumor cells. There are two main approaches used to design replication-competent adenoviruses in a tumor/tissue-specific manner: 1) the restrictive expression of the replication-essential viral proteins E1A, E1B, and E4 in target cells using tissue- or tumor-specific promoters and 2) the deletion of replication-critical genes. In this study, we constructed a tumor-specific, replication-competent adenovirus that was active in survivin-expressing cells. In other words, the survivin promoter was utilized to select the therapeutic targets. However, the survivin promoter was only 2- to 4-fold greater than the basal, TATA-mediated promoter in terms of transcription initiation activity and was thus unable to elicit a sufficient tumor cell killing effect by adenoviral lysis. We therefore further modified the adenovirus early E3 region to overexpress the adenovirus death protein (ADP), which resulted in enhanced killing activity. As shown in Figure 5, the replication competency that was mediated by the survivin promoter was lower than that of the wild-type E4 and E1 promoters by approximately 10-fold. However, the killing efficacy of Ad5/35E1apsurvivinE4 was comparable to the wild-type virus in all of the bladder cancer cells tested. It is conceivable that because this recombinant adenovirus markedly overexpressed ADP proteins, it was able to induce apoptosis in cancer cells as efficiently as the wild-type adenovirus. These results also suggest that weak promoters may be utilized for constructing therapeutic adenoviruses as long as they are target-specific.

Most of the studies of E1A-based replication-competent adenoviruses have demonstrated a loss of specificity because some of the promoters showed leaky *E1A* expression in non-target cells. As a result, dual control of the *E1A* and *E4* genes was shown to result in a greater degree of tissue-specific replication than with the *E1* gene alone. However, it can be difficult to detect two different tightly controlled tissue-specific promoters for any single tumor type. In addition, the two different promoters may result in promoter competition and a loss of tissue specificity. Moreover, the two juxtaposed, identical promoters may result in homologous recombination and the deletion of transgenes important for viral expression. In addition to the leakiness of the promoters, their application is restricted to only a narrow range of tumors because these promoters are only active in the type of cancer from which they were derived. The survivin promoter is therefore a good example of a promoter than can be applied to treat a variety of types of cells. In this study, we utilized the survivin promoter to construct a replication-competent adenovirus to target bladder cancer cells. One of the important features of the survivin promoter-driven adenovirus is that it can be used to target a variety of cancers. As shown in Figure 1, all of the bladder cancer cells tested appeared to express high levels of survivin mRNA transcripts. Furthermore, more than 60% of bladder cancer patients showed strong immunostaining results (data not shown). These results support the rationale that the survivin promoter may be highly active in bladder cancer cells and may therefore be utilized to target bladder cancer cells without harming normal cells.

Adenovirus vectors have been favored as a delivery vehicle for therapeutic genes because they are easy to construct, can be prepared at high titers, and are able to efficiently transduce a variety of cell types. Adenoviral vectors that are commonly used are based on Ad5, which belongs to subgroup C family of adenoviruses. CAR, which is recognized by the fiber knob protein of serotype 5, plays a crucial role in adenoviral infection of cancer cells. However, bladder cancer cell lines and tumors, especially those at an advanced state and high grade, frequently lose CAR expression, which limits the use of oncolytic adenoviruses for the treatment of bladder cancer. To overcome these limitations of Ad5 vectors, we used a different type of adenoviral vector, which was composed of Ad35. This vector belongs to subgroup B and recognizes human CD46, rather than CAR, as a cellular receptor for infection. Because CD46 is expressed in almost all human cells, Ad35 vectors demonstrate a broad tropism for human cells.

The survivin promoter, which is a *cis* regulatory element for survivin transcriptional expression, was shown to control the adenoviral early genes *E1A* and *E4* and to restrict viral replication to survivin-expressing cells. The recombinant adenovirus AdE1apsurvivinE4 was efficiently propagated and exhibited cytotoxic effects in all of the bladder cancer cell lines tested. Therefore, this adenovirus may serve as a prototype for further development of therapeutic adenoviruses for bladder cancer. Furthermore, future use of the AdE1apsurvivinE4 vector for clinical applications may generate a conditionally replicative adenoviral vector. This property of conditional replication would be expected to more efficiently target and kill bladder cancer cells compared to normal bladder cells and therefore contribute to the development of new treatments for bladder cancer.

## MATERIALS AND METHODS

### Cells

The bladder cancer lines T24, 253J, and J82 and the immortalized lung cell line L132 were maintained in RPMI 1640 media supplemented with 10% FBS and 1% penicillin/streptomycin (Invitrogen, Carlsbad, CA). Ku7-Luc cells (Caliper, Hopkinton, MA) were maintained in MEM supplemented with 10% FBS and 1% penicillin/streptomycin.

### RNA isolation and PCR

Total RNA was isolated using TRIzol reagent (Invitrogen). RT-PCR was performed using the Reverse Transcription System (Promega, Madison, WI). Primers for each specific gene were as follows: 5'-tag aac cat atc cca ggg aa -3' and 5'-gtc tac ctc ctt ttg aga ca -3' for survivin; 5'-aga agt cca cgc gtt gtg cat tgt-3' and 5'-cct aaa cca gct ggc caa aac-3' for E4orf6; and 5'-tgc acc acc aac tgc tta-3' and 5'-gga tgc agg gat gat gtt c-3' for GAPDH.

### Luciferase reporter assay

Each cell line was transiently co-transfected with 800 ng of pGL3psurvivin or pGL3TATA and 100 ng of pSV40renillaLuc for 16 h using Lipofectamine 2000 (Invitrogen) according to the manufacturer's protocol. Forty-eight hours after transfection, dual luciferase activity was measured in the cell lysates. Data represent the mean ± SD.

### Adenovirus construction

The construction strategy for Ad5/35E1apsurvivinE4 was developed in a previous study and underwent several subsequent modifications [39]. Briefly, adenovirus Ad5/35E1apsurvivinE4 was composed of following three cloning segments: 1) a shuttle vector, pAd1020*sfi*dA, which harbors a left-end inverted terminal repeat (ITR) and packaging signal (1-358 bp); 2) a modified adenovirus serotype 5 genome backbone vector termed E1bF5/35E4, which contains most of the Ad5 genome from the E1bTATA box to the E4TATA box but with the deletion of E4orf1-4; and 3) a shuttle vector termed p304*sfi* that contains the right-end ITR from 35,819 to 35,935 bp of the wild-type Ad5 genome. The left-sided CMV promoter-driven GFP expression cassette that was used to make pAd1020sfidA.CMV.GFP was described in a previous report [40]. pAd1020*Sfi*dA.CMV.GFP was then digested with the restriction enzyme *Sfi*I to remove CMV.GFP and the λ packaging signal. The survivin promoter (Invivogen, San Diego, CA) and *E1A* genes were inserted into p304*Sfi* to construct the intermediate vector p304*Sfi*psurvivinE1a. The third vector E1bF5/35E4 was prepared as described previously [39]. Three *Sfi*I-digested vectors were ligated, and the resultant cosmid was digested with the restriction enzyme *Pac*I and transfected into 911E4 cells to manufacture a large number of Ad5/35E1apsurvivinE4 virus particles.

### Western blot

For western blot analysis, the cells were lysed in RIPA buffer containing protease inhibitors (Sigma-Aldrich, St. Lois, MO). Lysates (20 μg) were resolved by SDS-PAGE and transferred to a PVDF membrane (Amersham Life Science). Membranes were first incubated with antibodies against E1A (Santa Cruz Biotechnology, Santa Cruz, CA) and then subsequently with a corresponding secondary antibody (Jackson Immuno Research Laboratories, West Grove, MA). Signals were visualized with a chemiluminescence reagent (Pierce, Rockford, IL).

### Viral replication assay

253J, T24, J82, and L132 cells (1 × 10^6^) were seeded into 6-well plates and subsequently infected with Ad5/35E4psurvivinE1a or Ad5wt. Each cell line was infected with 100 virus particles/cell, and the media was changed 24 h later. The fluorescent cells were examined daily under the microscope for up to 5 days. The supernatants containing the viral particles were harvested 5 days after infection, and the virus yield in the harvested virus supernatants was measured with titer assays. For the titer assays, HER911E4 cells (5 × 10^3^) were seeded into 96-well plates and infected with serial volume dilutions of the harvested supernatants, ranging from 1 to 10^−11^ μL per well. Doses of the produced viruses were defined as the dilution factor that caused a cytopathic effect in at least four wells of cells in one row of the 96-well plate on day 5. A tissue specificity index was obtained by dividing the dilution factor that caused a cytopathic effect for the therapeutic viruses by that for the wild-type virus.

### *In vitro* cytotoxicity assay

253J, T24, J82, Ku7-Luc, and L132 cells (5 × 10^3^) were seeded into 96-well plates 1 day prior to infection. The cells were infected with serial doses that ranged from MOIs of 50 to 5 × 10^−9^ for the AdE4psurvivinE1a and Ad5wt viruses. A row of eight wells was used for each serial dose, and the cells were examined under a light microscope on day 7. A killing activity index was calculated by dividing the dilution factor that caused a cytopathic effect for Ad5/35E4psurvivinE1a by that for Ad5wt. This value was expressed on a log_10_ scale such that a value of 1 indicated that Ad5/35E4psurvivinE1a had a 10-fold greater killing activity than the wild-type virus in a specific cell line.

### Animal studies

The orthotopic bladder cancer model was established as described in a previous study[41]. Ku7-Luc cells (1 × 10^6^ in PBS) were instilled intravesically via the urethra. After 4 days, the animals were checked by luminescence to confirm the presence of bladder tumors, and the mice were randomized into the two following groups: Ad35GFP and Ad5/35E4psurvivinE1a (n=5 tumor-bearing mice per group). Luminescence images were taken twice per week using the INVIVO Lumina instrument (Caliper).

### Statistical analysis

Comparisons between groups were made using the 2-tailed, unpaired t test.
